# HJURP regulates cell proliferation and chemo-resistance via YAP1/NDRG1 transcriptional axis in triple-negative breast cancer

**DOI:** 10.1038/s41419-022-04833-6

**Published:** 2022-04-22

**Authors:** Misha Mao, Yunlu Jia, Yongxia Chen, Jingjing Yang, Ling Xu, Xun Zhang, Jichun Zhou, Zhaoqing Li, Cong Chen, Siwei Ju, Linbo Wang

**Affiliations:** 1 Department of Surgical Oncology, Affiliated Sir Run Run Shaw Hospital, Zhejiang University, Hangzhou, 310000 Zhejiang China; 2 Biomedical Research Center and Key Laboratory of Biotherapy of Zhejiang Province, Hangzhou, 310000 Zhejiang China; 3 Department of Medical Oncology, the First Affiliated Hospital, Zhejiang University School of Medicine, Hangzhou, 310003 Zhejiang China

**Keywords:** Breast cancer, Cell signalling

## Abstract

Triple-negative breast cancer is still a difficult point in clinical treatment at present, and a deep study of its pathogenesis has great clinical value. Therefore, our research mainly focuses on exploring the progression of triple-negative breast cancer and determines the important role of the HJURP/YAP1/NDRG1 transcriptional regulation axis in triple-negative breast cancer. We observed significantly increased HJURP expression levels in triple-negative breast cancer compared to other subtypes. HJURP could affect the level of ubiquitination modification of YAP1 protein and then regulate its downstream transcriptional activity. Mechanistically, we found that YAP1 positively regulates NDRG1 transcription by binding the promoter region of the NDRG1 gene. And HJURP/YAP1/NDRG1 axis could affect cell proliferation and chemotherapy sensitivity in triple-negative breast cancer. Taken together, these findings provide insights into the transcriptional regulation axis of HJURP/YAP1/NDRG1 in triple-negative breast cancer progression and therapeutic response.

## Introduction

Triple-negative breast cancer (TNBC), the most malignant type of breast cancer among the four molecular types of breast cancer, typically lacks effective clinical treatment methods [1]. In particular, TNBC patients generally have a poor prognosis and high rates of systemic recurrence [2]. To date, endocrine therapy and targeted therapy such as trastuzumab do not benefit TNBC patients. There are few therapeutic options besides chemotherapy available to patients with aggressive TNBC due to a lack of targetable molecules. Therefore, it represents a pressing medical need for research based on the pathogenesis of TNBC and the availability of actionable targets.

The histone chaperone holiday junction recognition protein (HJURP) functions at the centromere level and has been proved to be required for CENP-A centromeric localization [3, 4]. Recent studies have shown that HJURP plays a dual role in the progression of glioblastoma, prostate cancer, and breast cancer [5–7]. For instance, suppression of HJURP induced cell senescence and abolished cell-cycle dynamics in glioblastoma [5]. HJURP increased the ubiquitination of CDKN1A via the GSK3β/JNK signaling pathway and decreased its stability, thus promoting prostate cancer cell proliferation [8]. In the field of breast cancer research, the expression of HJURP in breast cancer is significantly higher than that in normal tissues [9]. Previous work showed that HJURP is lower expressed in luminal A breast cancer than in other types of breast cancer and can distinguish between good and poor prognosis in luminal A breast cancer patients [10]. Additionally, high HJURP mRNA expression was also significantly associated with both shorter disease-free and overall survival and could predict the sensitivity of radiotherapy in breast cancer [9]. These suggested that HJURP might be a key regulator in breast cancer, but its roles in TNBC are not well understood.

To understand whether and how HJURP involves in the progression of TNBC, we performed a loss or gain function assay in TNBC cell lines and found the co-interaction between HJURP and YAP1 protein. More specifically, HJURP affected the ubiquitination level of YAP1 and caused the transcriptional regulation of its target gene NDRG1, thereby affecting cell proliferation and chemo-resistance of TNBC. This study provides theoretical support for finding relevant therapeutic targeting TNBC and has certain clinical guiding significance.

## Results

### HJURP modulates YAP1 protein level in TNBC cells

**Fig. 1:**
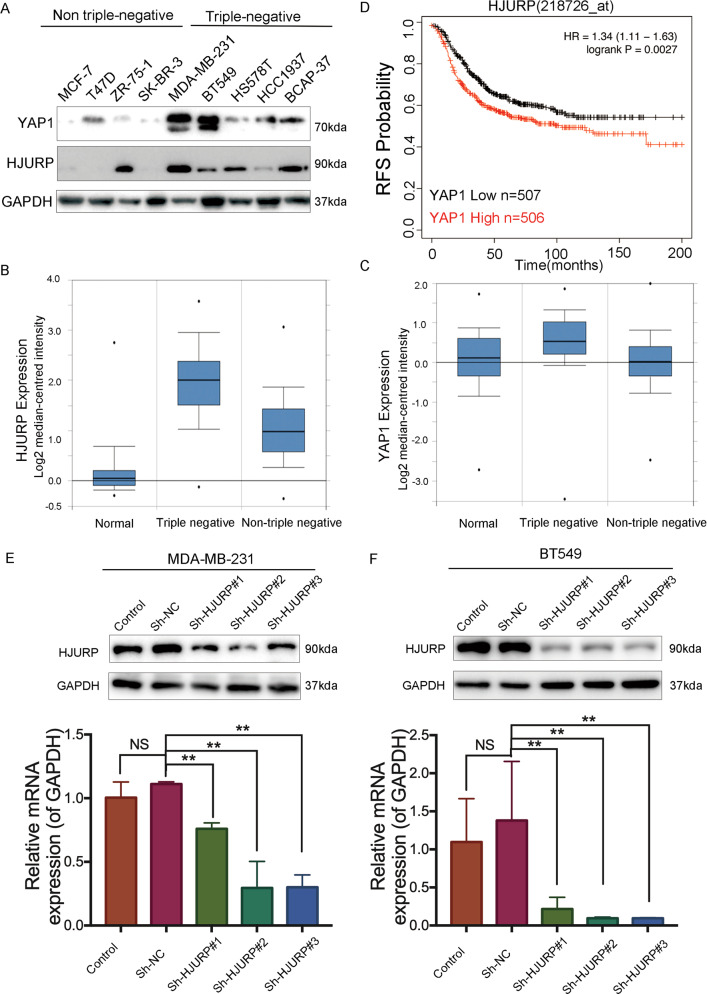
High HJURP and YAP1 expression in TNBC cells. **A** Western blot analysis of HJURP and YAP1 expression in breast cancer cell lines (MCF-7, T47D, ZR-75-1, SK-BR-3, MDA-MB-231, BT549, HS578T, HCC1937, and BCAP-37). **B**, **C** Oncomine database showed the expression of HJURP and YAP1 in normal breast tissues, triple-negative breast cancer, and non-triple-negative breast cancer. **D** Kaplan–Meier analyses RFS based on HJURP and YAP1 mRNA levels, using the KM-plotter breast cancer database (http://kmplot.com/analysis/). A median cutoff was chosen in the analysis. **E**, **F** The expression of HJURP in MAD-MB-231 cells and BT549 cells stably transfected with or without HJURP shRNAs was explored by western blot analysis and RT-PCR analysis. Data were presented as mean ± SD of three independent experiments. *p < 0.05; **p < 0.01; ns not significant.

**Fig. 2:**
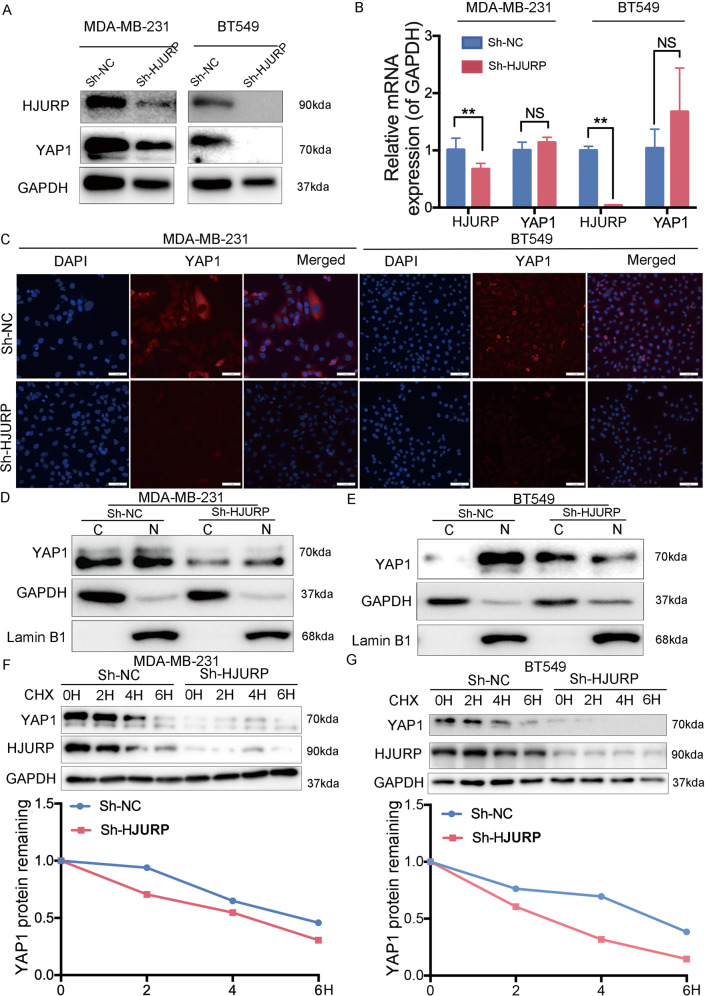
HJURP stabilizes and combines with YAP1 protein. **A** The protein level of HJURP and YAP1 was detected by western blot analysis in MDA-MB-231 cells and BT549 cells. **B** The mRNA level of HJURP and YAP1 was detected by RT-PCR analysis in MDA-MB-231 cells and BT549 cells. **C** Immunofluorescence detection of the DAPI-stained nuclei (blue), YAP1 expression (red), and merged fluorescence images illustrate predominant nuclear YAP1 localization. The scale bar represents 50 μm. **D**, **E** Western blot analysis of cytoplasmic and nuclear fractions of YAP1 in MDA-MB-231 cells and BT549 cells. **F**, **G** YAP1 protein was examined by western blot in MDA-MB-231 cells and BT549 cells with HJURP depletion after 72 h of transfection. The cells were treated with 10 μg/ml cycloheximide for the indicated times. The graph represents the quantification of the YAP1 protein levels. Data were presented as mean ± SD of three independent experiments. *p < 0.05; **p < 0.01; ns not significant.

To determine whether HJURP involves in the progression of TNBC, we firstly examined the expression level of HJURP in breast cancer cells. We revealed the expression of HJURP is significantly higher in TNBC than in non-TNBC cells (Fig. 1A). Interestingly, the expression of YAP1 protein was also significantly higher in TNBC cells, which is consistent with previously reported results [11]. Similar results were obtained from Oncomine databases. Both HJURP and YAP1 were higher in TNBC compared with normal breast and non-TNBC samples (Fig. 1B, C). These results indicated that HJURP may interact with YAP1 in TNBC. To test this hypothesis, we firstly performed Kaplan–Meier analyses using the online Kaplan–Meier–Plotter breast cancer database and the results showed that high expression of YAP1 mRNA level is associated with poor recurrence-free survival (RFS) in breast cancer patients with high HJURP expression (Fig. 1D). To validate the underlying mechanism, we selected MDA-MB-231 and BT549 cell lines for further exploration (Fig. 1E, F). After knocking down HJURP, the protein level of YAP1 expression was significantly decreased (Fig. 2A), but the mRNA level of YAP1 did not change (Fig. 2B). Next, we tested the cellular localization of YAP1 protein after HJURP depletion. Immunofluorescence localization (Fig. 2C) and Cytoplasmic and nuclear fractionation assay (Fig. 2D, E) indicated that the nuclear localization of YAP1 is significantly reduced after knocking down HJURP. Collectively, these results indicated that HJURP can inhibit YAP1 from entering the nucleus, thereby inhibiting its transcriptional activity.

### HJURP regulates YAP1 ubiquitination

**Fig. 3:**
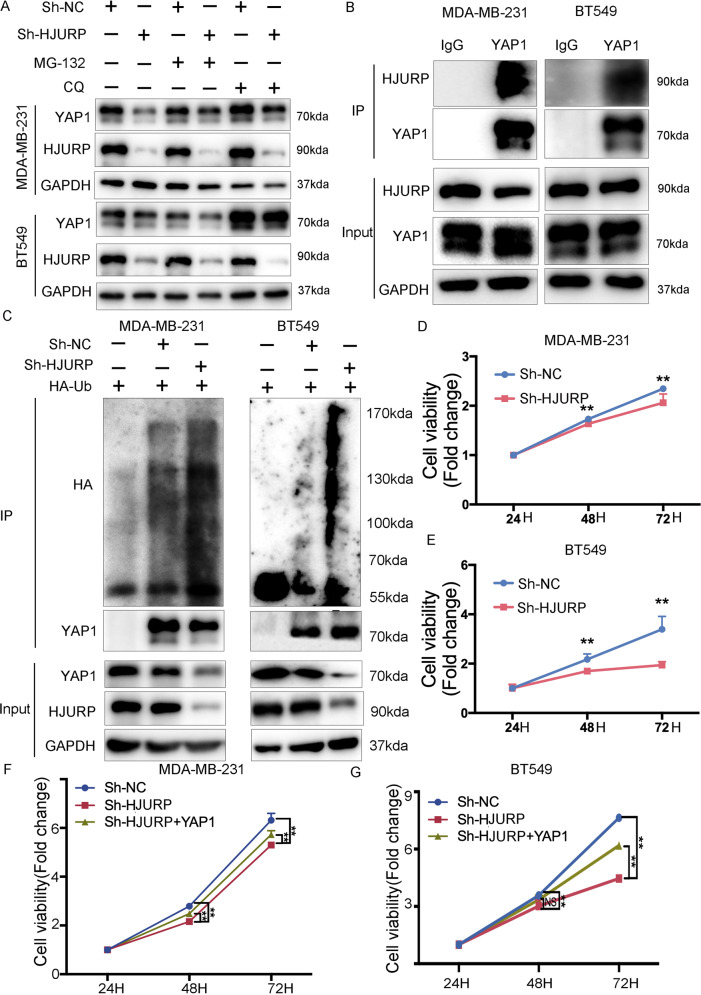
HJURP inhibits YAP1 ubiquitination. **A** Western blot analysis of YAP1 expression in MDA-MB-231 cells and BT549 cells with or without HJURP depletion in presence of proteasome inhibitor MG-132 (10 μM, 6 h) or lysosome inhibitor chloroquine (10 μM, 24 h). **B** Endogenous HJURP was analyzed by immunoprecipitation with YAP1 antibody in MDA-MB-231 cells and BT549 cells. **C** MDA-MB-231 cells and BT549 cells stably expressing Sh-HJURP were transfected with HA-Ub plasmid 48 h. Ubiquitination of YAP1 was analyzed by immunoprecipitation with YAP1 antibody and confirmed by western blot analysis. **D**, **E** Cell proliferation of MDA-MB-231 cells and BT549 cells stably expressing Sh-NC or Sh-HJURP was then determined by the CCK-8 assay at 24,48, and 72 h, respectively. **F**, **G** MDA-MB-231 cells and BT549 cells stably expressing Sh-NC or Sh-HJURP were transfected with the Flag vector or Flag-YAP1 and then seeded. Cell proliferation was then determined by the CCK-8 assay at 24,48, and 72 h, respectively. Data were presented as mean ± SD of three independent experiments. *p < 0.05; **p < 0.01; ns not significant.

To further investigate the impact of HJURP on YAP1 activity, we examined the effect of HJURP on YAP1 stability in MDA-MB-231 and BT549 cells treated with cycloheximide (CHX), an inhibitor of protein synthesis. We found that YAP1 stability is reduced after depletion of HJURP (Fig. 2F, G). As shown in Fig. 3A, HJURP could decrease YAP1 protein level, which could be minimized with the presence of the lysosome inhibitor Chloroquine but not proteasome inhibitor MG-132. Further immunoprecipitation experiments suggested that HJURP can bind with YAP1 protein (Fig. 3B). We then detected the effect of HJURP on the ubiquitination of YAP1 protein. The ubiquitination-based immunoprecipitation showed that HJURP inhibits YAP1 overall poly-ubiquitination (Fig. 3C). Therefore, the above experimental results suggested that HJURP binds to YAP1 protein and then regulates the stability and cellular distribution of YAP1, thereby promoting the ubiquitination level of YAP1.

### HJURP-regulated YAP1 activity promotes cell growth and reduces doxorubicin sensitivity in TNBC cells

**Fig. 4:**
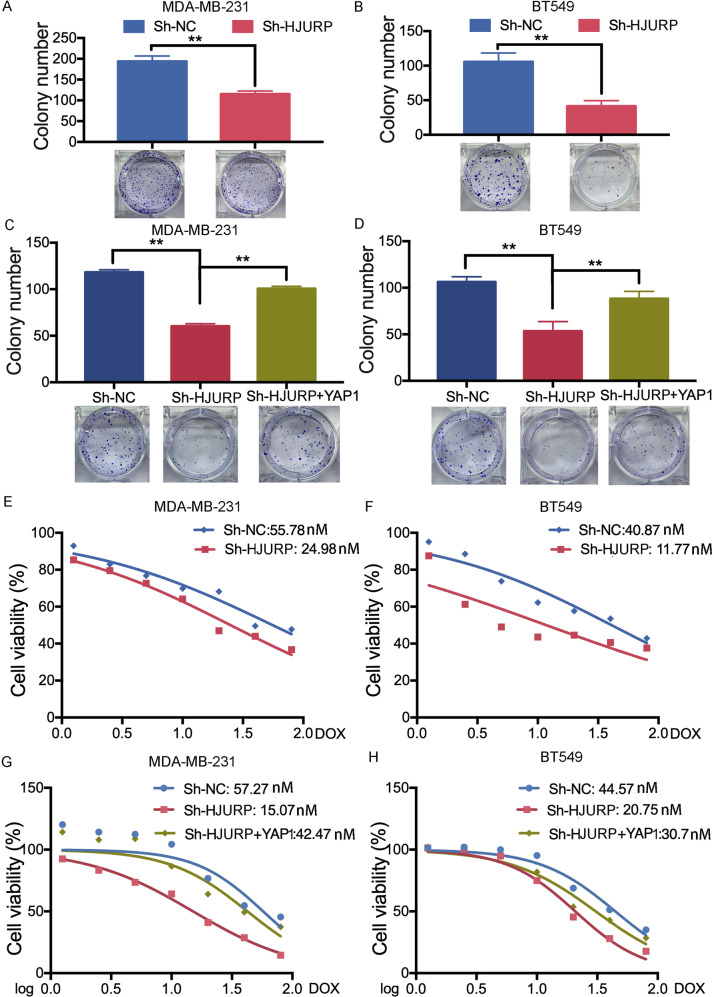
HJURP/YAP1 axis regulates TNBC cell proliferation and doxorubicin response. **A**, **B** MDA-MB-231 cells and BT549 cells stably expressing Sh-NC or Sh-HJURP were allowed to form colonies in a fresh medium for 14 days. **C**, **D** MDA-MB-231 cells and BT549 cells stably expressing Sh-NC or Sh-HJURP with or without YAP1 overexpression were allowed to form colonies in a fresh medium for 14 days. **E**, **F** MDA-MB-231 cells and BT549 cells stably expressing Sh-NC or Sh-HJURP were treated with doxorubicin at different concentrations for 48 h. Cell viability was detected by the CCK-8 assay. **G**, **H** MDA-MB-231 cells and BT549 cells stably expressing Sh-NC or Sh-HJURP with or without YAP1 overexpression were treated with doxorubicin at different concentrations for 48 h. Cell viability was detected by the CCK-8 assay. Data were presented as mean ± SD of three independent experiments. *p < 0.05; **p < 0.01; ns not significant.

To investigate the effects of the HJURP/YAP1 axis on the malignant phenotypes of TNBC cells, we performed a functional assay in TNBC cells after HJURP silencing. Knockdown of HJURP significantly suppressed cell growth determined by the CCK-8 assay (Fig. 3D, E). And this inhibition was reversed by overexpressing YAP1 (Fig. 3F, G). Cell colony formation assay showed that cell growth reduction by HJURP silencing can be reversed again after YAP1 overexpression (Fig. 4A–D). Additionally, depletion of HJURP could induce doxorubicin sensitivity in MDA-MB-231 and BT549 cells (Fig. 4E, F). YAP1 overexpression abolished HJURP knockdown-induced chemotherapy sensitivity in MDA-MB-231 and BT549 cells (Fig. 4G, H). Collectively, these findings demonstrated that HJURP/YAP1 signaling pathway is responsible for TNBC proliferation and response to chemotherapy.

### HJURP/YAP1- axis promotes NDRG1 transcription

**Fig. 5:**
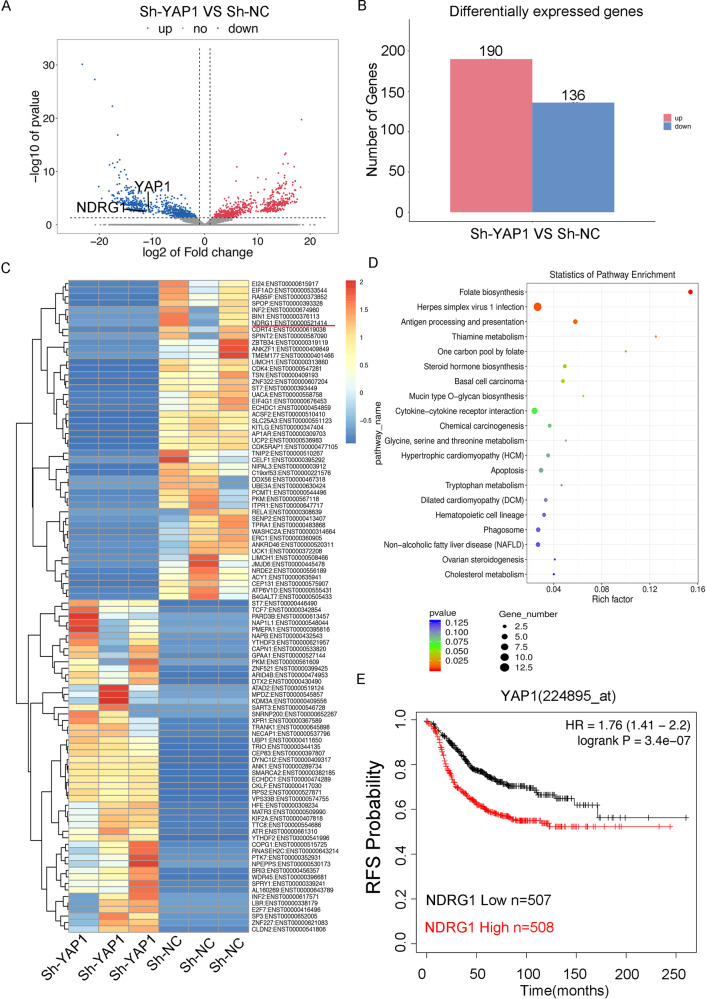
HJURP/YAP1 axis regulates NDRG1 transcriptional level. **A**, **B** RNA sequence analysis was performed in MDA-MB-231 cells stably expressed Sh-NC or Sh-YAP1 (*n* = 3). It showed differential transcriptomic expression. **C** The Heatmap diagram with transcripts RNAs in MDA-MB-231 cells stably expressing Sh-NC or Sh-YAP1 (*n* = 3). The color scale at the right illustrates the relative expression level of each transcript across all samples; red and blue are expression levels above and below the mean, respectively. White is the mean expression level. **D** Top changed pathways in Sh-YAP1 groups versus Sh-NC groups were shown. **E** Kaplan–Meier analyses RFS based on YAP1 and NDRG1 mRNA levels, using the KM-plotter breast cancer database (http://kmplot.com/analysis/). A median cutoff was chosen in the analysis.

**Fig. 6:**
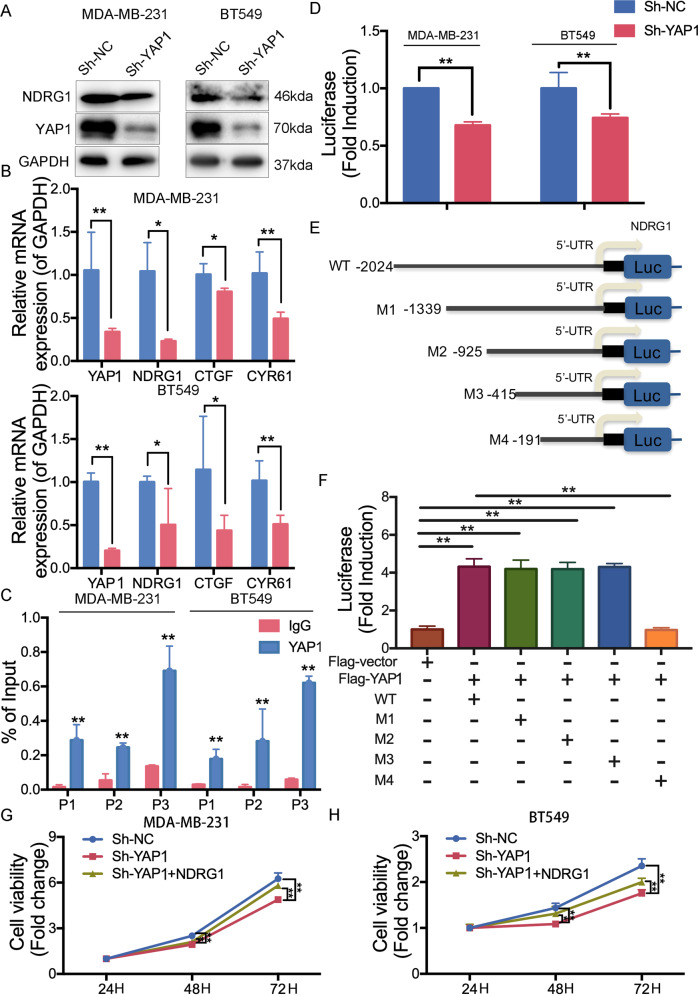
HJURP/YAP1 axis induces NDRG1 transcription. **A**, **B** Western blot analysis and RT-PCR analysis of NDRG1 expression in MDA-MB-231 cells and BT549 cells stably expressing Sh-NC or Sh-YAP1. **C** Enrichment of YAP1 in the NDRG1 promoters was measured by ChIP experiments in MDA-MB-231 cells and BT549 cells. **D** Promoter activities of NDRG1 in MDA-MB-231 cells and BT549 cells stably expressing Sh-NC or Sh-YAP1. **E**, **F** Different promoter activities of NDRG1 in 293 T transfected with Flag vector or Flag-YAP1, as measured by a dual-luciferase assay. **G**, **H** MDA-MB-231 cells and BT549 cells stably expressing Sh-NC or Sh-YAP1 were transfected with the Flag vector or Flag-NDRG1 and then seeded. Cell proliferation was then determined by the CCK-8 assay at 24, 48, and 72 h, respectively. Data were presented as mean ± SD of three independent experiments. *p < 0.05; **p < 0.01; ns not significant.

In order to further analyze the regulatory network of the HJURP/YAP1 pathway in TNBC, we performed RNA-sequencing after knocking down YAP1 in MDA-MB-231 cells (Supplementary Fig. 1A, B). The results showed a total of 326 gene expression changes, of which 136 genes were up-regulated after knocking down the YAP1, and 190 genes were downregulated (Fig. 5A, B). The Go enrichment was shown in Supplementary Fig. 2C. We found that the expression of NDRG1 transcripts is significantly decreased in the YAP1 knockdown group (Fig. 5C, D). Previous research also indicated that mRNA expression of NDRG1 is relatively higher in TNBC than that in other types of breast cancer cells [12]. Additionally, Kaplan–Meier meta-analyses showed that a high expression of NDRG1 mRNA level is specifically significantly associated with RFS in breast cancer patients with high YAP1 expression (Fig. 5E). Depletion of YAP1 in MDA-MB-231 and BT549 cells decreased the NDRG1 expression (Fig. 6A, B). 6A, B and YAP1 target genes (CTGF and CYR61) were previously validated to be determined the transcriptional activity of YAP1(Fig. 6B). We then demonstrated that YAP1 can bind to the promoter region of the NDRG1 gene determined by the CHIP results (Fig. 6C). We constructed the promoter region of the NDRG1 gene to be inserted into the pezx-pg04.1vector. Luciferase reporter system assays also showed that YAP1 can positively regulate NDRG1 transcription (Fig. 6D). In order to further clarify the binding site, we constructed different truncations of the NDRG1 promoter region and found that only the 0–191 region of the NDRG1 promoter can cause changes in its transcription levels (Fig. 6E, F). Taken together, these results indicated that HJURP/YAP1 regulatory axis is important for the transcription activation of NDRG1.

### HJURP/YAP1/NDRG1 pathway relates to the tumor growth of TNBC in vivo and in vitro

**Fig. 7:**
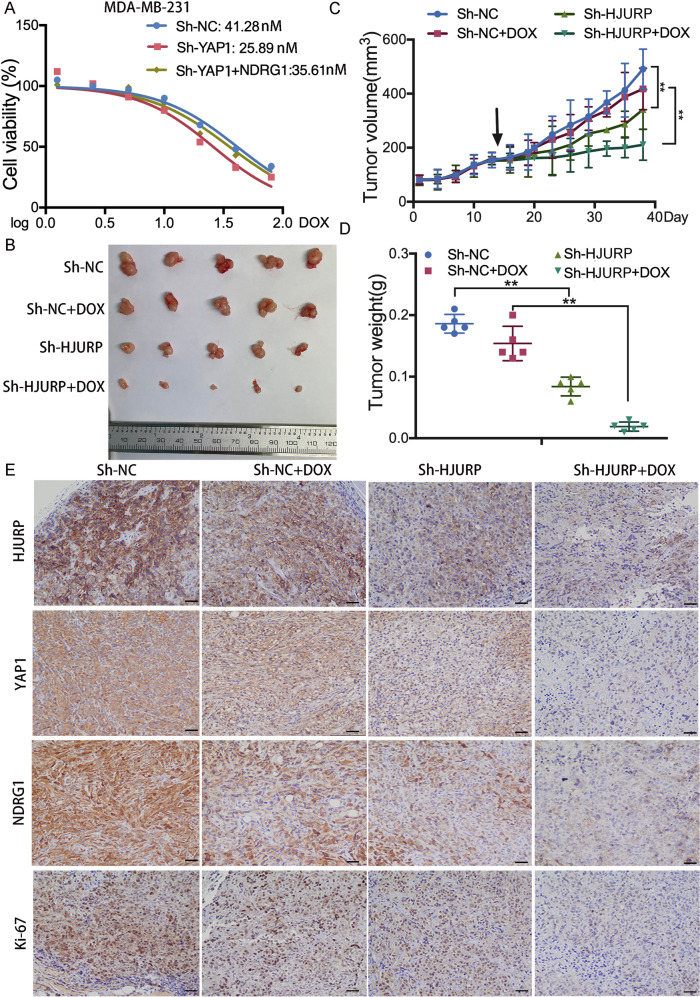
HJURP depletion suppresses tumor growth via downregulating YAP1 and NDRG1 expression in vivo. **A** MDA-MB-231 cells stably expressing Sh-NC or Sh-YAP1 with or without NDRG1 overexpression were treated with doxorubicin at different concentrations for 48 h. Cell viability was detected by the CCK-8 assay. **B** Images of tumor in xenograft-bearing mice. **C** Tumor volume (every three days) of xenograft-bearing mice in the four groups. **D** Tumor weight (Day 24 after drug treatment) of xenograft-bearing mice in the four groups. **E** IHC staining of tumor tissues shows the expression of HJURP, YAP1, NDRG1, and Ki-67. The scale bar represents 50 μm. Data were presented as the mean ± SD *P < 0.05, **P < 0.01.

To understand the roles of the HJURP/YAP1/NDRG1 axis underlying the formation of TNBC cells, we knocked down the YAP1 and cell growth was decreased, while this inhibition was reversed by overexpressing NDRG1 (Fig. 6G, H). The colony number was significantly reduced in the YAP1 knockdown group and increased again after overexpressing NDRG1 (Supplementary Fig. 1D, E). Additionally, NDRG1 overexpression abolished YAP1 knockdown-induced chemotherapy sensitivity both in MDA-MB-231 and BT549 cells (Fig. 7A and Supplementary Fig. 2A). We further investigated the role of this pathway with in vivo assay. Mouse xenograft assays were performed by injecting BALB/c nude mice with MDA-MB-231 cells stably depleted of HJURP (Fig. 7B). We found that depletion of HJURP led to decreased tumor size and was more sensitive to doxorubicin (Fig. 7C, D), suggesting that HJURP activation is important for TNBC tumor growth and chemotherapy response in vivo. The immunohistochemical images showed HJURP/YAP1/NDRG1 axis promotes TNBC tumor growth (Fig. 7E). Taken together, these results supported a crucial role of the HJURP/YAP1/NDRG1 pathway in TNBC in vivo and in vitro.

## Discussion

Traditional molecular classification of breast cancer includes luminal A, luminal B, HER2-positive, and TNBC. At present, with the advancement of diagnosis and treatment methods, hormone-positive endocrine therapy and HER2-positive targeted therapy have greatly improved the survival rate of breast cancer patients. However, TNBC still lacks effective therapeutic targets and prognostic indicators. Therefore, we explored the pathogenesis of TNBC and its regulatory network.

Here, we firstly reported the underlying regulatory mechanisms of HJURP in TNBC. The expression of HJURP is relatively higher in TNBC cells compared with other types of breast cancer cells, which is consistent with previous work [13]. Loss of function experiments identified that HJURP promotes TNBC cells proliferation and decreases doxorubicin sensitivity. In order to clarify its regulatory system, we then tested the expression of YAP1, which is an important transcriptional regulator and participates in important biological roles in tumors, especially in TNBC [14–16]. For example, YAP1 could induce basal-like breast cancer stemness [17]. Aurora A can affect the transcription of the YAP1 gene and its phosphorylation level in TNBC [11]. Because HJURP and YAP1 are both highly expressed in basal-like breast cancer, YAP1 as a transcriptional factor plays an important role in TNBC. HJURP was positively related to YAP1 in TNBC. Our results further showed that after the down-expression of HJURP, the activity of YAP1 protein decreased without a change in its mRNA level. HJURP could bind with YAP1 protein by using immunoprecipitation experiments. The results suggested that HJURP affects YAP1 expression at the posttranscriptional level. Considering the posttranslational regulation, ubiquitin is an evolutionarily conserved protein that post-translationally marks proteins for degradation and has been shown to be necessary for various protein functions [18]. Proteins marked by ubiquitination are usually trafficked to the proteasome or lysosome for degradation [18, 19]. YAP1 has been reported to be ubiquitinated by other proteins [20]. For example, PARK2 promoted YAP1 ubiquitination through proteasome-dependent degradation in esophageal squamous carcinoma [21]. RACO-1 promoted proteasome-dependent YAP1 degradation via inducing YAP1 K48-linked poly-ubiquitination and inhibiting YAP1 K63-linked poly-ubiquitination [22]. Additionally, HJURP can also regulate the ubiquitination of various proteins [8, 23]. Our results suggested that HJURP reflects YAP1 protein stability and regulates the level of ubiquitinated YAP1 protein. Moreover, HJURP inhibited YAP1 ubiquitination via lysosome-dependent degradation. Additionally, cell proliferation assays indicated that the inhibition of cell growth and doxorubicin sensitivity after knocking down the HJURP can be rescued by overexpressing YAP1. The HJURP/YAP1 pathway may act as a potential barrier to TNBC development and drug resistance.

To better characterize the HJURP/YAP1 signaling axis’ mechanism in TNBC, we reduced the level of YAP1 in MDA-MB-231 cells and performed RNA-sequencing. Notably, we noticed that the transcript level of NDGR1 is closely related to the change of YAP1 expression. NDRG1 is a member of the NDRG protein family consisting of NDRG1-4, which are evolutionarily well conserved [24]. And NDRG1 has been proved to participate in the apoptosis process of a variety of tumors [25–27]. Until now, NDRG1 has been implicated in divergent processes in breast cancer, complicating the interpretation of its function [28]. NDRG1 reflects neutral lipid metabolism thus promoting breast cancer proliferation, and the mRNA expression is relatively higher in TNBC cells [12]. Silencing of YAP1 caused downregulation of NDGR1 transcription level. We further confirmed the regulation of the YAP1 and NDGR1 at the transcriptional level through luciferase experiments and CHIP experiments. We then conducted the different regions of the NDRG1 promoter to identify the combination of the specific regions that realizes the effect on its transcriptional activity. The results showed that the 0–191 region of the NDRG1 promoter is the main responsible area for YAP1 transcriptional regulation. At the same time, we also found that the HJURP/YAP1/NDRG1 regulatory axis affects cell proliferation and drug resistance in TNBC. Cell proliferation assay indicated that YAP1 can promote cell proliferation and induce doxorubicin resistance and these inductions are reverted again after overexpressing NDRG1. We further performed tumor-forming animal experiments in nude mice that showed that tumor volume is significantly reduced after downregulation of HJURP especially when treated with doxorubicin. Altogether, these results showed that HJURP/YAP1/NDRG1 pathway regulated cell proliferation and doxorubicin resistance of TNBC in vitro and in vivo.

Taken together, our data point to the potential genetic mechanisms that can dysregulate TNBC progression. We found that HJURP can combine with YAP1 protein and then reflect its ubiquitination via lysosome-dependent degradation in TNBC. Additionally, HJURP/YAP1 regulatory axis could induce the transcriptional level of NDRG1. HJURP/YAP1/NDRG1 pathway is important for the physiological processes of TNBC. Therefore, our study determined a new regulation axis in TNBC and provided important theoretical support for TNBC treatment targets and related predictors.

## Materials and methods

### Cell culture

The human breast cancer cell lines (T47D, ZR-75-1, BT549, HCC1937, and BCAP-37) were maintained in RPMI 1640 medium supplemented with 10% fetal bovine serum and 5% glutamine. MCF-7 and HS578T cell lines were maintained in Dulbecco’s Modified Eagle’s Medium supplemented with 10% fetal bovine serum and 0.01 mg/ml bovine insulin. SK-BR-3 cell line was maintained in McCoy’s 5 A Medium supplemented with 10% fetal bovine serum. The MDA-MB-231 cell line was maintained in Leibovitz’s L-15 Medium supplemented with 10% fetal bovine serum and incubated cultures at 37 °C without CO_2_. All cell lines except MDA-MB-231 grow in a humid atmosphere containing 5% CO_2_ at 37 °C.

### Chemicals and antibodies

Chlorhexidine(#HY-B1248), Chloroquine(#HY-17589A), MG-132(#HY-13259), and Doxorubicin(#HY-121309) were purchased form Med Chem Express. The primary antibody HJURP (#712465, 1:1000) was purchased from Thermo Fisher Scientific. The primary antibodies YAP1 (#14074, 1:1000), Lamin B1 (#13435, 1:1000), and NDRG1(#9485, 1:1000) were purchased from Cell Signaling Technology. GAPDH (1:1000) antibodies were purchased from Santa Cruz Biotechnology.

### Overexpression and knockdown of genes

Overexpressing plasmid or shRNA of indicated genes were transfected into cells using Lipofectamine 3000(Invitrogen, Carlsbad, CA) (Thermo Fisher) according to the manufacturer’s instructions which were described previously [29]. Then the efficacy was confirmed 48–72 h later.

### Cell viability assays

Triple-negative breast cancer cells were seeded onto a 96-well culture plate at different times with or without the drug. Then, cell viability was evaluated using the CCK-8 assay. The absorbance was measured at 450 nm by using a BioTek ELx800 absorbance microplate reader.

### Cell colony formation assay

Triple-negative breast cancer cells were seeded into a six-well plate and incubated for 14 days. Then, the cells were fixed with 4% polyoxymethylene at room temperature for 10 min. After being washed with PBS three times, the cells were stained with crystal violet solution at room temperature for 30 min. The excess crystal violet was removed and washed with water for three times.

### Western blot analysis

Western blots analysis was performed as previously described [29]. Nuclear fractionation used a nuclear protein extraction kit (Beyotime, China). Three independent experiments were performed.

### RNA isolation and quantitative real-time PCR

These experiments were performed as described previously [30]. In general, total RNA was extracted from cells and tissues using TRIzol (Invitrogen, Carlsbad, CA) reagent. All samples were amplified thrice in real-time, and the expression was normalized to GAPDH. The following primer sequences were used:

GAPDH forward 5′-TGACTTCAACAGCGACACCCA-3′

GAPDH reverse 5′-CACCCTGTTGCTGTAGCCAAA-3′

HJURP forward 5′-GATTCAAAAAGCGGTGAGGTCG-3′

HJURP reverse 5′-AGTCACACGTACATCCCTTCC-3′

YAP1 forward 5′-TAGCCCTGCGTAGCCAGTTA-3′

YAP1 reverse 5′-TCATGCTTAGTCCACTGTCTGT-3′

Cyr61 forward 5′-GGTCAAAGTTACCGGGCAGT-3′

Cyr61 reverse 5′-GGAGGCATCGAATCCCAGC-3′

CTGF forward 5′-ACCGACTGGAAGACACGTTTG-3′

CTGF reverse 5′-CCAGGTCAGCTTCGCAAGG-3′.

RNA-sequencing (GSE131369) was performed by LC-Bio Technology CO. Ltd., Hangzhou, China.

### Immunofluorescence (IF) and immunohistochemical staining (IHC)

Immunofluorescence analyses and immunohistochemical staining were performed as previously described [29]. For immunofluorescence assay, cells were incubated with YAP1 antibody (1:1000) at 37 °C for 1 h, followed by Alexa 555-conjugated (red) goat anti-rabbit antibody (1:1000) (Multisciences, Hangzhou, China). And for the immunohistochemical staining assay, the slices were stained with HJURP (1:100), YAP1 (1:500), NDRG1(1:1000), and Ki-67 (1:1000).

### Immuno‐precipitation assay (IP)

Triple-negative breast cancer cells were harvested and lysed with NP-40 buffer for 30 min on ice. Then cell lysates were centrifuged at high speed while the remaining 10% of the sample was used to prepare inputs. The remaining cell lysates were immunoprecipitated using specific antibodies and then added to 30 μL agarose A + G. And the mixture was co-incubated at 4 °C overnight on a rotary shaker. The samples were washed with PBS three times and visualized by western blot.

### Ubiquitination assay

Cells were transfected with the indicated plasmids and treated with 10 μM chloroquine for 12 h or 10 μM MG-132 for 6 h before harvesting. Cells were harvested and lysed with NP-40 buffer for 30 min on ice. Then cell lysates were centrifuged at high speed while the remaining 10% of the sample was used to prepare inputs. The remaining lysates were subjected to immunoprecipitation with IgG or YAP1 antibody at 4 °C overnight. The samples were collected and washed with PBS three times and the ubiquitination of endogenous YAP1 was detected by western blot.

### Luciferase assays

The activities of Gaussia luciferase and secreted alkaline phosphatase in a dual-reporter system were measured 48 h post-transfection with an NDRG1 promoter-reporter plasmid using the Secrete-Pair Gaussia Luciferase Assay Kit (LF031, GeneCopoeia) following the manufacturer’s instructions. The luminometer was used to acquire the activities. Each experiment was repeated at least three times independently.

### Chromatin immunoprecipitation (ChIP)

Chromatin immunoprecipitation was performed using the SimpleChIP^®^ Enzymatic Chromatin IP Kit (Magnetic Beads) (Cell Signaling Technology, 9003) following the instructions of the manufacturer. Chromatin was used for immunoprecipitation with anti-YAP1, anti-histone 3, and a normal rabbit- IgG antibody. ChIP-enriched DNA was measured by using real-time PCR. The primer sequences were used:

F1 5′-GAGCCGACCCACAACCC-3′

R1 5′-CACCCCTTCCCCGCTC-3′

F2 5′- AGGGGACTGCAGAGCCGA-3′

R2 5′- CGCGCGGCGGGCGCCCA-3′

F3 5′- CCCTACGACTGCTTGCGCAA-3′

R3 5′- CGCCCACTGGAGCCGCCG-3′.

### Tumor xenografts in nude mice

Twenty BALB/c female nude mice (4 weeks old) were purchased from Shanghai Laboratory Animal Center and housed in a specific pathogen-free environment according to the Ethics Committee for Animal Studies of Zhejiang University (Hangzhou, China). Sh-NC or Sh-HJURP MDA-MB-231 cells (1 × 10 × 6 per mouse) were injected into the right sub-axillary region. Mice were monitored and were measured twice per week using a slide caliper including tumor length (L), and width (W). The tumor sizes were measured twice a week. Tumor volume (mm^3^) = π/6 × length × width^2^. When tumors reached a volume of ~150 mm^3^, Sh-NC or Sh-HJURP mice were randomly allocated into groups and treated with or without doxorubicin (5 mg/kg every week) via intraperitoneal injection for 24 days. Mice were then sacrificed and some tumor tissues were fixed with 10% paraformaldehyde for immunohistochemical analysis.

### Statistical analysis

Data were analyzed in Excel and graphed in GraphPad Prism 7.0 software (San Diego, CA, USA). The comparisons between multiple groups were performed using multiple comparisons by one-way ANOVA. Comparisons between groups were performed using Student’s *t*-test and an unpaired two-tailed Student’s *t*-test was used to compare mean data. Data were independently obtained from at least three experiments. The results are presented with statistical significance or *P* value (*p < 0.05; **p < 0.01; NS indicated not statistically significant).

## Supplementary information

Supplementary figure legends

aj-checklist

Supplementary Figure 1

Supplementary Figure 2

Supplementary Figure 3

## Data Availability

The data that supports the findings of this study are available in the supplementary materials of this article.
