# Supplementary material for: HJURP regulates cell proliferation and chemo-resistance via YAP1/NDRG1 transcriptional axis in triple-negative breast cancer
Source: Cell Death Dis. 2022 Apr 22;13(4):396. doi: 10.1038/s41419-022-04833-6 (PMC9033877; doi:10.1038/s41419-022-04833-6)
Supplement: Supplementary file 1 — Supplementary figure legends [file 41419_2022_4833_MOESM1_ESM.docx]

**Supplementary Figure 1.** (A) and (B) The expression of YAP1 in MAD-MB-231cells and BT549 cells stably transfected with or without YAP1 shRNAs was explored by western blot analysis and RT-PCR analysis. (C) Go enrichment of genes in MDA-MB-231 cells stably expressing Sh-NC or Sh-YAP1. (D) MDA-MB-231 cells stably expressing Sh-NC or Sh-YAP1 with or without NDRG1 overexpression were allowed to form colonies in fresh medium for 14 days. (E) BT549 cells stably expressing Sh-NC or Sh-YAP1 with or without NDRG1 overexpression were allowed to form colonies in fresh medium for 14 days. Data were presented as mean ± SD. of three independent experiments. *p < 0.05; **p < 0.01; ns, not significant.

**Supplementary Figure 2**. (A) BT549 cells stably expressing Sh-NC or Sh-YAP1 with or without NDRG1 overexpression were treated with doxorubicin at different concentrations for 48 h. Cell viability was detected by the CCK-8 assay. Data were presented as mean ± SD. of three independent experiments. *p < 0.05; **p < 0.01; ns, not significant.
