# Supplementary figures and images for: HJURP regulates cell proliferation and chemo-resistance via YAP1/NDRG1 transcriptional axis in triple-negative breast cancer
Source: Cell Death Dis. 2022 Apr 22;13(4):396. doi: 10.1038/s41419-022-04833-6 (PMC9033877; doi:10.1038/s41419-022-04833-6)

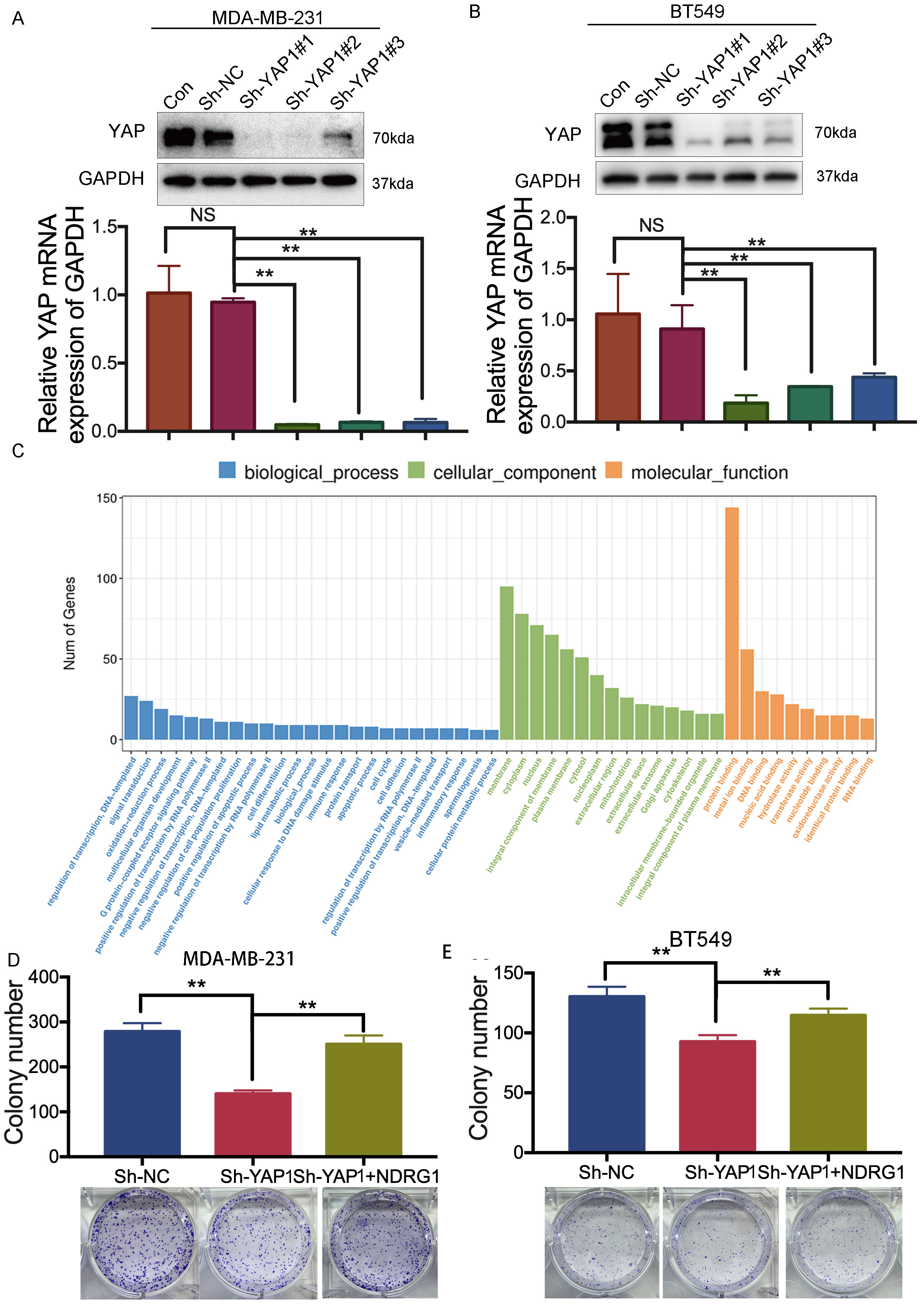

Supplement: Supplementary file 3 — Supplementary Figure 1 [file 41419_2022_4833_MOESM3_ESM.jpg]

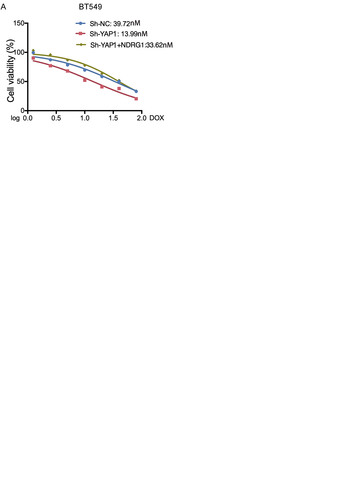

Supplement: Supplementary file 4 — Supplementary Figure 2 [file 41419_2022_4833_MOESM4_ESM.jpg]

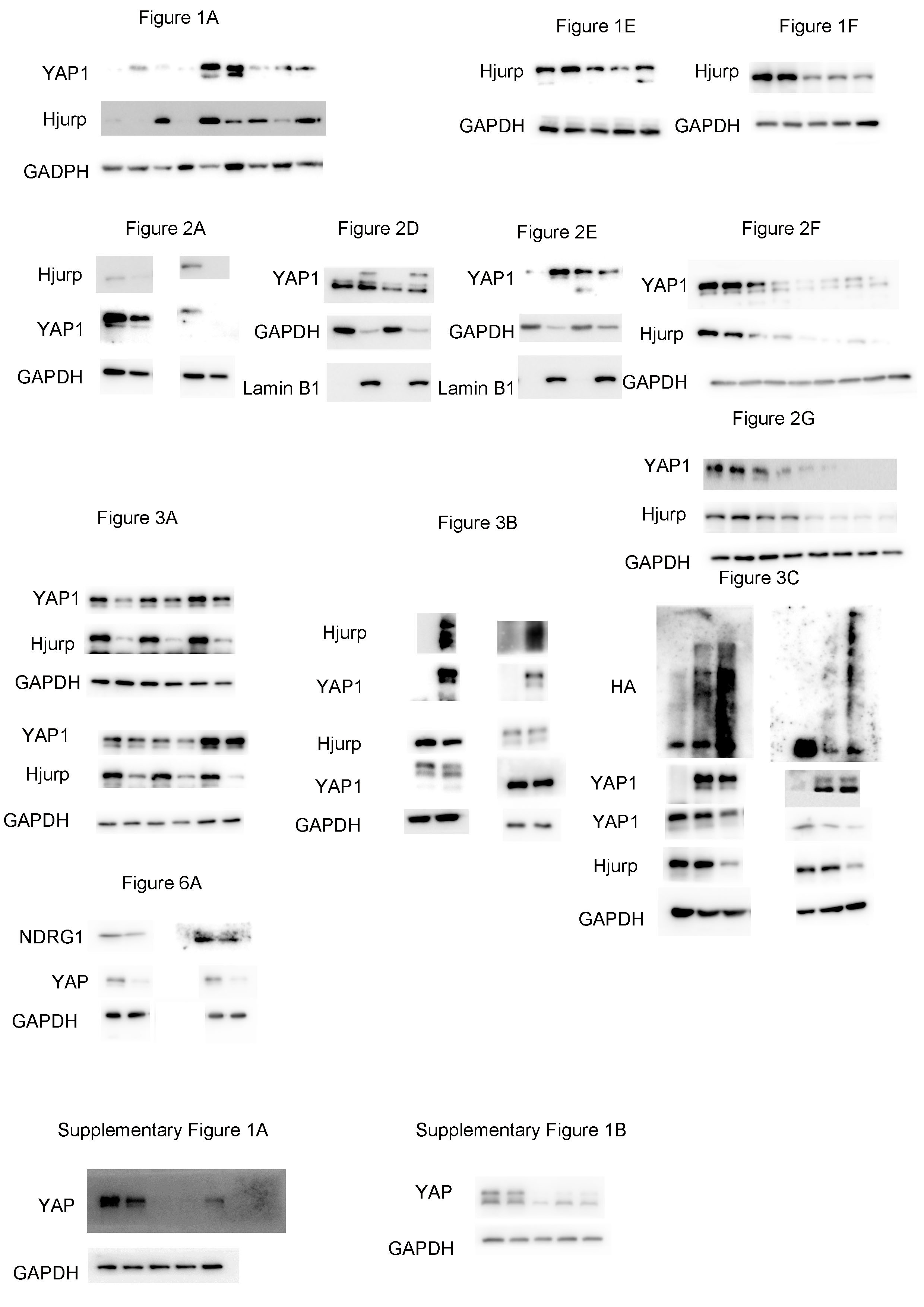

Supplement: Supplementary file 5 — Supplementary Figure 3 [file 41419_2022_4833_MOESM5_ESM.jpg]
